# Co-purification of the GroEL chaperone during outer membrane vesicle purification: insights from Aeromonas salmonicida subsp. salmonicida

**DOI:** 10.1099/mic.0.001558

**Published:** 2025-04-28

**Authors:** Maude F. Paquet, Steve J. Charette

**Affiliations:** 1 Institut de biologie intégrative et des systèmes (IBIS), Université Laval, Quebec City, Quebec, Canada; 2 Département de biochimie, de microbiologie et de bio-informatique, Faculté des sciences et de génie, Université Laval, Quebec City, Quebec, Canada

**Keywords:** *Aeromonas salmonicida* subsp. salmonicida, Amicon® column, electron microscopy, GroEL, optiprep gradient, outer membrane vesicles (OMVs), purification

## Abstract

Outer membrane vesicles (OMVs) are naturally produced by Gram-negative bacteria and originate from their outer membrane. They can be extracted using ultracentrifugation or ultrafiltration using concentration columns, followed by purification with a density gradient. However, these methods may co-purify contaminants with similar physical properties. Several studies have identified GroEL, a chaperonin, as a major protein in OMV preparations. Using *Aeromonas salmonicida* subsp. *salmonicida* as a model, we detected GroEL by mass spectrometry and observed it in transmission electron microscopy images as separate from OMVs. As a cytoplasmic protein complex, GroEL is more likely a contaminant resulting from bacterial lysis during growth rather than an intrinsic OMV component. The model *A. salmonicida* subsp. *salmonicida* proved valuable in reaching this conclusion because it produces high levels of extracellular GroEL and low amounts of OMVs. This study emphasizes the need for caution when interpreting the presence of GroEL in OMV preparations and highlights the importance of rigorous purification methods to ensure OMV purity.

## Introduction

Outer membrane vesicles (OMVs) are formed by the outward budding of the outer membrane of Gram-negative bacteria. The OMVs range in size from 10 nm to 400 nm [1,4]. Since OMVs originate from the bacterial membrane, their composition is closely similar to their parent membrane, though some proteins appear to be enriched in the OMVs [5,6]. OMV production is a natural process that serves multiple functions, including phage protection, antibiotic resistance, excretion of toxic components like misfolded proteins or as transporters of toxins [7,10]. The study of OMVs is an expanding field, partly due to their potential as vaccines against bacterial diseases [11,12].

## OMVs from *Aeromonas salmonicida* subsp. *salmonicida*

To investigate the diverse roles of OMVs, they must first be extracted and purified. Two commonly used extraction methods are ultracentrifugation and ultrafiltration concentration using Amicon® columns. Amicon® columns retain molecules or complexes with a molecular weight greater than 100 kDa [2]. Ultracentrifugation separates substances based on density, pelleting those with higher densities. Following extraction, OMVs can be further purified using a density gradient called Optiprep [13], which is a 60% iodixanol solution. The gradient is composed of varying concentrations of iodixanol that go from 10% to 30%. OMVs accumulate at a specific point within the gradient, based on their density [13].

**Fig. 1.**
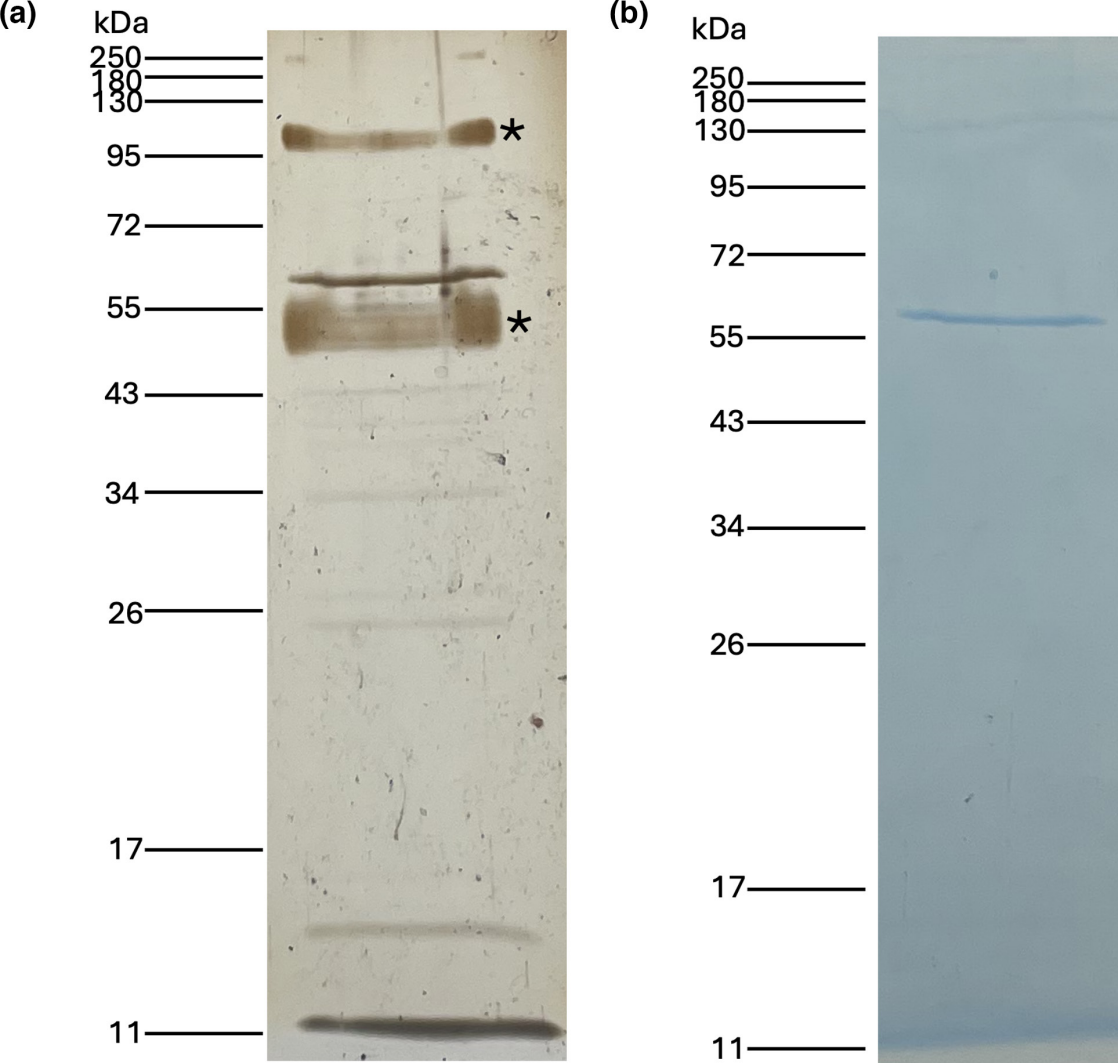
Comparison between silver and Coomassie blue staining of proteins on SDS-PAGE gels. Proteins from the OMV extraction of *A. salmonicida* subsp. *salmonicida* SHY15-2939 were visualized using (**a**) silver nitrate staining or (**b**) Coomassie blue staining. Samples were heated at 95 °C for 7 min and β-mercaptoethanol was added prior to the loading on the gel. Both gels contained 12% acrylamide, and the migration was at 120 V for 1 h 20 min.

Once OMVs are obtained and purified, we can perform various analyses such as protein characterization. The first step is to analyse the proteins associated with OMVs by SDS-PAGE. To visualize protein bands, two commonly used staining methods are silver nitrate and Coomassie blue. Silver staining is 50–100 times more sensitive than the Coomassie blue coloration [14]. Fig. 1 illustrates this difference in sensitivity by showing the same OMV extraction from the strain SHY15-2939 of the aquatic pathogen *A. salmonicida* subsp. *salmonicida* [15]. The SDS-PAGE gel stained with silver nitrate clearly reveals more bands than the one stained with Coomassie blue. The two groups of bands marked with asterisks on the silver nitrate gel are not proteins but are most likely LPS. Silver staining can detect LPS, and those two groups of bands have been well characterized in the literature as corresponding to LPS of *A. salmonicida* subsp. *salmonicida* [16,17]. Therefore, only the bands in the lower portion of the silver-stained gel (below 43 kDa) and the band at ~60 kDa are likely OMV-associated proteins. In contrast, the Coomassie-blue-stained gel reveals only a single band, also at ~60 kDa.

## Co-purification of GroEL

Thereafter, the 60 kDa band was excised and analysed by mass spectrometry at the Proteomics Platform at the Quebec Genomics Centre, with data processed using the software Scaffold v.5.3.3. The protein was identified as GroEL, a chaperonin involved in the proper folding of misfolded proteins [18]. GroEL is a large protein complex with a tetradecameric structure composed of two identical heptameric rings of seven 57 kDa subunits totalizing a complex of about 800 kDa [18]. This conformation gives GroEL a cylindrical shape with an internal cavity. To function effectively, GroEL requires its co-chaperonin GroES, a smaller complex composed of a single heptameric ring, with each subunit weighing 10 kDa [18]. The complex GroEL–GroES is localized in the cytoplasm of bacteria [18].

It was questionable whether GroEL could be the major OMV protein because it is an intracellular protein rather than an outer membrane protein. However, multiple studies have also identified GroEL in their OMV extraction for other bacterial species. Table 1 summarizes 11 studies that have reported GroEL in OMVs isolated from 10 different bacterial species.

**Table 1.** Compilation of the detection of GroEL in different studies

		OMV method of analysis*				
Bacteria studied	Method of extraction and/or purification	SDS-PAGE	TEM	Mass spectrometry or N-terminal sequencing	Immunogold labelling of GroEL	Reference
*Shigella sonnei*	Centrifugation 4,000 g	**+**	**+**	**+**		[19]
*Escherichia coli*	Ultracentrifugation 208,000 g+Optiprep	**+**	**+**	**+**		[22]
*Acinetobacter baumannii*	Ultracentrifugation 150,000 g+100 kDa hollow fibre membrane +sucrose gradient	**+**	**+**	**+**		[36]
*Campylobacter jejuni*	Ultracentrifugation 100,000 g+trichloroacetic acid precipitation	**+**	**+**	**+**	**+**	[21]
*Francisella novicida*	Ultracentrifugation 125,000 g		**+**	**+**		[24]
*Cronobactersp*.	Ultracentrifugation 150,000 g	**+**	**+**	**+**		[23]
*Helicobacter pylori*	Amicon® column 100 kDa	**+**	**+**	**+**		[37]
*Escherichia coli*	Amicon® column 100 kDa +ultracentrifugation 75,000 g		**+**	**+**		[20]
*Francisella noatunensis* subsp. *orientalis*	Ultracentrifugation 100,000 g	**+**	**+**	**+**		[38]
*Aggregatibacter actinomycetemcomitans*	Ultracentrifugation 85,000 g+Optiprep	**+**		**+**		[39]
*Myxococcus xanthus*	Ultracentrifugation 140,000 g	**+**	**+**	**+**		[40]

*:A plus symbol (+) denotes that the procedure was conducted in the specified study.

The interpretation of GroEL’s presence varies among studies. Some authors suggested that GroEL is likely a contaminant originating from lysed cells [19,21], while others proposed that GroEL is probably associated with the vesicle membrane [22,24]. A common factor among these studies is the similarity in the OMV extraction/purification protocols. They all used ultracentrifugation (with or without the Optiprep purification) or the Amicon® column. These methods could possibly lead to the co-concentration of molecules with a similar density to the OMVs in the Optiprep gradient, while the Amicon® column would retain molecules larger than 100 kDa alongside the OMVs.

Before extracting OMVs, bacteria must be cultivated and grown in an appropriate medium. During growth, some cells may lyse during the exponential growth phase by autolysis, with lysis increasing further in the stationary phase [25]. The contents of those lysed cells are released into the medium. When OMVs are extracted, for example, using the Amicon® column with *A. salmonicida* subsp. *salmonicida*, any released material with a molecular weight greater than 100 kDa will be concentrated along with the OMVs. Since GroEL is a large protein complex of 800 kDa, if it is released from the cells, it will also be retained in the Amicon® column. GroEL is the most abundant chaperone in *E. coli* and one of the major proteins in the cytosol [26], further supporting the hypothesis that GroEL is co-purified with OMVs rather than being an intrinsic OMV component.

Lindmark *et al.* tested this hypothesis using immunogold labelling [21]. They used an antiserum that recognized GroEL in the OMV preparations obtained from *Campylobacter jejuni*. Their results showed that GroEL particles were on the outside of the OMVs rather than in direct association with them, which led the authors to suggest that the presence of GroEL in OMV preparations is likely due to contamination during the extraction process.

**Fig. 2.**
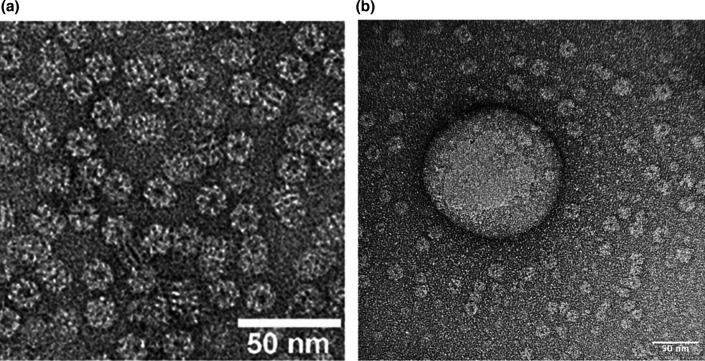
Comparison between GroEL particles and unknown materials from an OMV extraction of *A. salmonicida* subsp. *salmonicida* in TEM. (a) Purification of GroEL particles. This picture is from [27]. (**b)** OMV extraction from *A. salmonicida* subsp. *salmonicida* using an Amicon® column and the Optiprep gradient. The scale bar on both images is 50 nm.

The phenomenon of co-purification is further supported in the present study by transmission electron microscopy (TEM) images of OMVs from *A. salmonicida* subsp. *salmonicida*. Fig. 2 compares an image of purified bacterial GroEL particles done in a separate study [27] with unknown materials present in an OMV preparation from *A. salmonicida* subsp. *salmonicida*. The OMVs were concentrated and purified using the Amicon® column followed by an Optiprep gradient. In panel (**a**), the heptameric rings of GroEL, with their characteristic central cavity, are clearly visible. In panel (**b**)**,** an OMV of ~150 nm is surrounded by numerous unidentified particles measuring 15–20 nm. These unknown particles closely resemble GroEL particles because they have a shape and a central cavity similar to the GroEL in panel (**a**). GroEL measures 15 nm in height and 14 nm in width [28], consistent with the unknown particles in *A. salmonicida* subsp. *salmonicida*. The presence of GroEL throughout the TEM field, and not preferentially associated with the OMV, supports the hypothesis that GroEL is not an OMV protein, but rather a contaminant from lysed cells.

The production of OMVs by *A. salmonicida* subsp. *salmonicida* is relatively low, resulting in a high GroEL-to-OMV ratio. This elevated proportion makes it easier to conclude that GroEL is not specifically associated with OMVs. In contrast, previous studies with a lower GroEL-to-OMV ratio faced greater difficulty in distinguishing whether GroEL was genuinely co-purified or intrinsically linked to OMVs. In this context, *A. salmonicida* subsp. *salmonicida* serves as a valuable model for addressing this question.

Other studies have suggested that GroEL may be attached to the cell wall and not only present in the cytoplasm [29,31]. However, given its primary function as a chaperonin, it is unlikely that GroEL is actively exported and deliberately attached to the outer surface of the cell. A more plausible explanation for this observation in these studies is that GroEL particles released from lysed cells adhere non-specifically to the cell walls of other intact bacteria.

Ribosomes are also large protein complexes of about 2,500 kDa that are abundant in bacteria [32]. Given their size and prevalence, one might question why ribosomes are neither observed in TEM images nor identified in the mass spectrometry results of OMV preparations. One possible explanation is that ribosomes require Mg^2+^ ions to maintain their structural stability. If the concentration of Mg^2+^ is below 10 mM, the ribosomal subunits will dissociate [33]. Since this concentration is not maintained outside the bacterial cell in OMV preparations, the ribosomes are unlikely to persist. Additionally, extracellular RNases can degrade ribosomal RNA, potentially altering the structure of the ribosome and further preventing their detection [34].

TEM observations are also essential for drawing accurate conclusions. Many of the TEM images presented in the articles summarized in Table 1 are uninformative, with insufficient magnification to clearly distinguish GroEL structures. Also, some images contain an excessive number of OMVs, which may obscure smaller co-purified particles such as GroEL, making them difficult to detect. As mentioned above, using *A. salmonicida* subsp. *salmonicida*, with its low amount of OMVs, as a model in this study, combined with the opportunity to work with a high-performance 200 kV TEM, allowed us to make observations that were instrumental in reaching these conclusions.

## Conclusion

To conclude, GroEL is not directly associated with the OMVs in Gram-negative bacteria, but it is likely co-purified with them during the OMV extraction. Its presence in OMV preparations suggests that GroEL has a similar density to OMVs, so GroEL and OMVs get co-purified in the same Optiprep fraction. Additionally, its molecular weight allows it to be retained in the Amicon® column. This implies that GroEL remains stable outside the bacterial cell and that GroEL particles should be eliminated from the OMV extraction to ensure OMV purity. One possible approach to achieving this is through immunoprecipitation using magnetic beads [35]. By coupling an antibody specific to GroEL to magnetic beads, GroEL could be selectively removed from OMV preparations, improving their purity for downstream analyses.

## Abbreviations

OMVs: outer membrane vesicles
TEM: transmission electron microscopy
